# Heart Rate Dynamics after Combined Strength and Endurance Training in Middle-Aged Women: Heterogeneity of Responses

**DOI:** 10.1371/journal.pone.0072664

**Published:** 2013-08-27

**Authors:** Laura Karavirta, Madalena D. Costa, Ary L. Goldberger, Mikko P. Tulppo, David E. Laaksonen, Kai Nyman, Marko Keskitalo, Arja Häkkinen, Keijo Häkkinen

**Affiliations:** 1 Department of Biology of Physical Activity, University of Jyväskylä, Jyväskylä, Finland; 2 Division of Interdisciplinary Medicine and Biotechnology, Beth Israel Deaconess Medical Center, Harvard Medical School, Boston, Massachusetts, United States of America; 3 Wyss Institute for Biologically Inspired Engineering at Harvard University, Boston, Massachusetts, United States of America; 4 Department of Exercise and Medical Physiology, Verve Research, Oulu, Finland; 5 Department of Medicine, Kuopio University Hospital, Kuopio, Finland; 6 Department of Physiology, University of Eastern Finland, Kuopio, Finland; 7 Central Finland Central Hospital, Jyväskylä, Finland; 8 Department of Health Sciences, University of Jyväskylä, Jyväskylä, Finland; University of Adelaide, Australia

## Abstract

The loss of complexity in physiological systems may be a dynamical biomarker of aging and disease. In this study the effects of combined strength and endurance training compared with those of endurance training or strength training alone on heart rate (HR) complexity and traditional HR variability indices were examined in middle-aged women. 90 previously untrained female volunteers between the age of 40 and 65 years completed a 21 week progressive training period of either strength training, endurance training or their combination, or served as controls. Continuous HR time series were obtained during supine rest and submaximal steady state exercise. The complexity of HR dynamics was assessed using multiscale entropy analysis. In addition, standard time and frequency domain measures were also computed. Endurance training led to increases in HR complexity and selected time and frequency domain measures of HR variability (*P*<0.01) when measured during exercise. Combined strength and endurance training or strength training alone did not produce significant changes in HR dynamics. Inter-subject heterogeneity of responses was particularly noticeable in the combined training group. At supine rest, no training-induced changes in HR parameters were observed in any of the groups. The present findings emphasize the potential utility of endurance training in increasing the complex variability of HR in middle-aged women. Further studies are needed to explore the combined endurance and strength training adaptations and possible gender and age related factors, as well as other mechanisms, that may mediate the effects of different training regimens on HR dynamics.

## Introduction

Cardiac interbeat interval time series are conventionally analyzed using linear time and frequency domain measures of heart rate variability (HRV) to indirectly examine cardiac autonomic function [1]. However, heart rate (HR) dynamics also exhibit complex fluctuations that are thought to reflect nonlinear interactions among multiple control mechanisms [2]. These complex HR fluctuations typically demonstrate multiscale variability that conventional measures based on mean, variance or Fourier spectrum techniques alone cannot fully capture [3]. Methods derived from the theory of nonlinear dynamics, such as, multiscale entropy may, therefore, provide complementary information about the structure of these time series.

HR complexity has been proposed as an index of integrated cardiac regulation; the higher the complexity of the system the greater its functionality [4]. Physiological aging is associated with the loss of HR complexity [5], [6]. Aging in conjunction with reduced volume and intensity of physical activity, leads to inevitable progressive impairment, not only in cardiac autonomic function, but also in cardiorespiratory fitness and muscular strength. Endurance training improves cardiac vagal modulation and cardiorespiratory fitness, even in the elderly [7], [8], but has minor effects on neuromuscular performance [9]. Strength training, on the other hand, increases muscular strength in aging men and women [10]. However, the findings regarding the effects of strength training on HR dynamics are not consistent; some studies have reported a positive change [11], [12] while others have found no change [13], [14].

Both strength and endurance training produce training mode-specific cardiovascular and neuromuscular adaptations [15], [16] and thus, strength and endurance training should be performed simultaneously to improve all the essential aspects of physical performance and health. However, the distinct physiological adaptations to these two different types of training stimuli may under certain conditions impair optimal training adaptations. Therefore, the purpose of this study was to examine the effects of combined strength and endurance training compared with those of endurance training or strength training alone on HR complexity and HRV in middle-aged women.

## Methods

### Subjects

The study plan was approved by the Ethics Committee of the University of Jyväskylä. The participants were informed about the design of the study and possible risks and discomforts related to the measurements, and all participants signed a written informed consent. Healthy untrained 40 to 65 year old women were recruited for the intervention by advertising in newspapers and through e-mail lists. Subjects underwent an examination of general health and a resting electrocardiogram (ECG), administered by a physician. Subjects without cardiovascular or musculoskeletal disorders, diabetes, or medications known to influence cardiovascular or neuromuscular performance continued in the study. The subjects who passed the medical examination performed a clinical exercise test to voluntary exhaustion with ECG and blood pressure monitoring under the supervision of a physician. Subjects with evidence of cardiovascular or musculoskeletal problems were excluded from the study.

Three subjects withdrew after the randomization into the study groups due to personal reasons, and 102 subjects continued participation in the intervention. Six women did not complete the training or control period due to diagnosed type 2 diabetes (n = 1) or personal reasons (n = 5). Furthermore, six subjects were excluded from the further analysis due to the noise or excessive ectopic beats in RR interval time series (see the section below on the analysis of RR interval time series). Thus, the data are reported from 90 subjects. The characteristics of the final subject groups at baseline are presented in table 1.

**Table 1:** Age, height, weight, BMI, VO_2_peak and MVC at baseline.

	E (n = 26)	S (n = 26)	SE (n = 21)	C (n = 17)
Age	52 (7)	52 (8)	49 (6)	52 (8)
Height, cm	163 (7)	164 (7)	164 (5)	166 (6)
Weight, kg	66.9 (9.7)	66.3 (9.7)	66.2 (9.1)	66.5 (7.5)
BMI, kg·m^−2^	25.1 (2.7)	24.7 (3.0)	24.7 (3.3)	24.1 (2.4)
VO_2_peak, ml·kg^−1^·min^−1^	25.3 (5.2)	25.9 (5.4)	27.7 (4.6)	26.1 (5.8)
MVC, N	1990 (428)	1952 (441)	1987 (521)	1844 (324)

Values given as mean (standard deviation). E; Endurance training group; S, Strength training group; SE, Combined strength and endurance training group; C, Control group; BMI, body mass index; VO_2_peak, peak oxygen uptake; MVC, maximal voluntary contraction during isometric bilateral leg extension.

### Experimental Design

The subjects were randomized into four groups: endurance training (E), strength training (S), combined strength and endurance training (SE) or controls (C). The measurements were performed at baseline and after 10 and 21 weeks of training. Subjects in the control group were measured at baseline and after 21 weeks. This study was part of a larger project, and the data on physical performance [17], body composition [9] and serum hormone concentrations [18] have previously been published.

### Cardiorespiratory Fitness Test

A graded maximal exercise test to volitional exhaustion was performed on a mechanically braked bicycle ergometer (Ergomedic 839E, Monark Exercise AB, Sweden) with simultaneous electrocardiographic (ECG) and blood pressure monitoring. The test was supervised by a physician. Exercise intensity was increased by 20 W every 2^nd^ minute starting with 50 W, and pedalling frequency was sustained at 60 rpm throughout the test. Maximal testing was preceded by a warm-up period of five minutes at the same exercise intensity (50 W) as the first actual test grade. Respiratory parameters were measured continuously breath by breath (SensorMedics Vmax229; SensorMedics Corporation, Yorba Linda, CA). Peak oxygen uptake (VO_2_peak) was determined as the highest minute average of VO_2_ during the test.

### Measurement of Maximal Strength

Maximal voluntary contraction (MVC) during isometric bilateral leg extension was measured on a dynamometer [19] in a seated position with a knee angle of 107° and a hip angle of 110°. Subjects were instructed to generate maximum force as rapidly as possible against the force plate for a duration of 2–4 s. Subjects performed a minimum of three trials, and the trial with the highest peak force was selected for further analysis. The force signal was low-pass–filtered (20 Hz) and analyzed (Signal Software version 2.15; Cambridge Electronic Design Ltd., Cambridge, UK).

### RR Interval Recording at Rest and during Exercise

**Figure 1:**
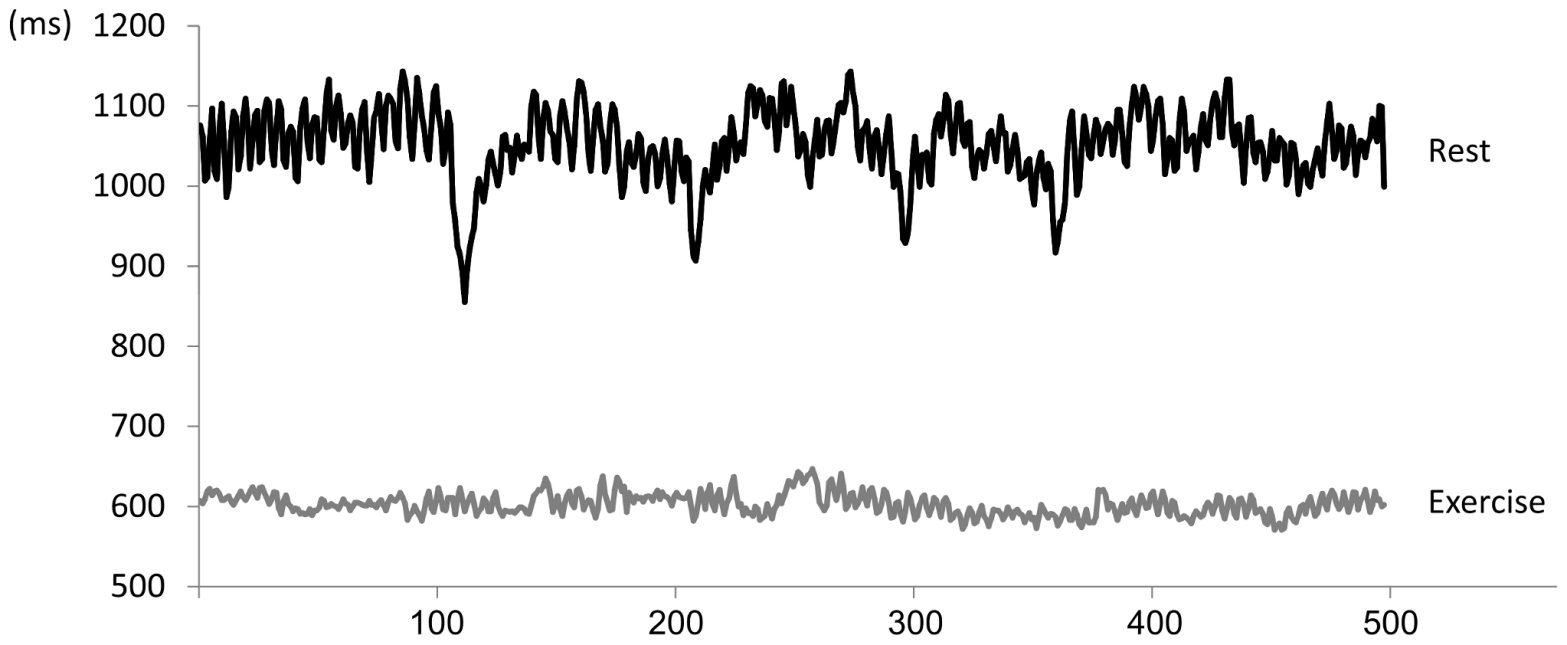
An example of RR interval time series (500 intervals) measured during supine rest and during low intensity exercise.

RR intervals were recorded throughout the cardiorespiratory fitness test and analyzed from the 5-minute steady state part of the exercise at exercise intensity of 50 W starting after two minutes of the initiation of exercise (Fig. 1). Additionally, RR intervals were recorded at rest, a minimum of 72 hours after the cardiorespiratory fitness test. The recordings were done between 7∶00 and 9∶00 a.m., after overnight fasting. The measurement took place in a quiet room. The RR intervals were recorded in a supine position for ten minutes. The breathing frequency was spontaneous. RR intervals were collected for further analysis with Polar S810i HR monitors by using online recording with infrared interface and Polar Precision Performance software (Polar Electro Ltd, Kempele, Finland). The Polar S810i HR monitor has been validated for the recording both at rest and during exercise with an accuracy better than 2 ms when compared to an ECG method [20], [21]. Noise and ectopic heart beats were identified and eliminated by an automatic process in the software; furthermore, the data were then inspected visually for possible artifacts. RR interval time series containing more than 15% noise or ectopic beats were excluded from further analysis. The precise number of RR intervals analysed was dependent on the subjects’ HR and was approximately 600 for both exercise and resting conditions.

### Analysis of the RR Interval Time Series

For the time domain analysis, filtered RR intervals from normal sinus rhythm (NN intervals) and the corresponding average HR were computed. Standard deviation of all NN intervals (SDNN) was calculated as the square root of variance. Analysis of power spectral density provides information about how the variance (power) is distributed as a function of frequency [2]. Frequency domain variables high frequency power (0.15<HFP≤0.40 Hz) and low frequency power (0.04<LFP≤0.15 Hz) were analyzed using the Lomb periodogram [22]. Due to the short duration of the recordings very low frequency power was not included in the analysis. The assessment of HFP and LFP components was made in absolute values of power (ms^2^).

HR complexity was measured using the multiscale entropy (MSE) method, described in detail elsewhere [3], [6], [23], [24]. Briefly, this method quantifies the information content of a time series, measuring its multiscale structural richness. The algorithm comprises two steps: 1) a coarse-graining procedure to derive a set of time series, each of which represents the system’s dynamics at different time scales, and 2) the quantification of the degree of irregularity of each coarse-grained time series, which can be accomplished using an entropy measure such as sample entropy (SampEn) [3]. Technically, SampEn is the negative natural logarithm of an estimate of the conditional probability that sequences matching pointwise for *m* consecutive data points, within a tolerance *r*, also match for *m+1* data points. Here, we used *m* = 2 and *r* = 8 ms [25].

For the NN interval time series obtained at rest, a complexity index (CI) was computed by summing SampEn values for scales 1 to 5 (CI_1–5_); for the time series obtained during exercise, the complexity index included only scales of 1 to 2 (CI_1–2_) due to the shorter duration of these recordings (5 vs. 10 minutes).

The higher the complexity index, the higher the information content of the signal. Lowest complexity values, computed over a range of time scales, are observed for both highly periodic (predictable) signals and completely unpredictable (random) signals. Both classes of time series convey the lowest information content and reflect the lowest adaptability of a system.

### Endurance Training

Endurance training was carried out twice a week [26]. The HR levels for endurance training were determined based on respiratory parameters and blood lactate concentrations, as described in detail previously [27]. All training sessions were supervised, and HR monitoring was used. During the first 7 week period, the subjects trained on a bicycle ergometer for 30 min below the level of the aerobic threshold. Weeks 5–7 during the first period also included three training sessions during which the subjects were accustomed to the intensity above the aerobic threshold by a 10 minute interval in the middle of the sessions. During weeks 8–14, one weekly session of 45 min included a 10 min interval between the aerobic-anaerobic thresholds and a 5 min interval above the anaerobic threshold, in addition to a 15 min warm-up and a 15 min cool down below the aerobic threshold. The other weekly training session involved 60 min of cycling below the aerobic threshold. The focus of training during weeks 15–21 was to improve maximal endurance. One of the weekly sessions lasted for 60 min, which included two 10 min intervals between the aerobic-anaerobic thresholds, two 5 min intervals above the anaerobic threshold, and 30 min below the aerobic threshold. The other weekly session included 90 min cycling at a steady pace below the aerobic threshold.

### Strength Training

Strength training was carried out twice a week. All strength training sessions were supervised. The strength training program included 7–10 exercises that activated all of the main muscle groups. Every training session included two exercises for the leg extensors (leg press and knee extension), one exercise for knee flexors (leg curl), and one to two other exercises for the lower extremities (seated calf raise, hip abduction or adduction). For the upper body, each session included three to four exercises (bench press, biceps curl, triceps pull-down, lateral pull-down), and one to two exercises for the trunk (abdominal crunch, seated back extension). The overall intensity and amount of training increased progressively throughout the 21 week training period. [19], [26].

The training period was divided into three 7 week cycles to optimize strength gains and muscle hypertrophy. The focus of the first cycle was to accustom the subjects to the high intensity training and to improve muscle strength and muscle endurance using light loads (40–60% of 1RM) and a high number (12–20) of repetitions, and by performing 3 sets. The second cycle (weeks 8–14) was designed to produce muscle hypertrophy to further increase the total muscle mass/fat ratio by increasing the loads progressively up to 60–80% of the maximum, with 5–12 repetitions and 2–4 sets. To further enhance strength development and muscle hypertrophy during weeks 15–21, higher loads of 70–85% of 1RM together with 5–8 repetitions and 2–4 sets were used. In addition, approximately 20% of the leg press, knee extension and bench press exercises was performed with light loads of 40 to 50% of 1RM and 5–8 repetitions, to meet the requirements of a typical explosive strength training protocol. With the light loads, each repetition was executed as rapidly as possible [19]. Adherence of the required loads and repetitions during each training cycle was ensured by 10RM tests and careful supervision.

### Combined Strength and Endurance Training

The subjects in the combined group performed endurance training twice a week and strength training twice a week, performing a total of 4 training sessions per week as described in the preceding paragraphs [28].

### Statistical Analyses

The results are expressed as means and standard deviations or means and 95% confidence intervals. The assumptions for analysis of variance (ANOVA) (homogeneity of variance, sphericity, and normal distribution) were tested, and if the assumptions were not met, a natural logarithmic transformation was used. The effects of different training regimens over time were examined using ANOVA for repeated measures. The Pearson product-moment correlation coefficient was used to evaluate the relationships between variables. The critical level for statistical significance in all tests was set at 0.05. The statistical analyses were carried out using the PASW statistics 18.0 software for Windows (SPSS Inc., Chicago, IL).

## Results

The average training adherence was 98.0 (1.6) % in the endurance and 99.7 (2.2) % in the strength training sessions. There were no differences between groups in training adherence when comparing the adherence of strength training in the S and SE groups or the adherence of endurance training in the E and SE groups. All subjects completed a minimum of 90% of the total training volume. Furthermore, training groups were statistically similar at baseline in terms of age, height, weight, BMI, VO_2_peak, MVC and HRV indices.

Individual changes in physical performance have been previously reported [17]. Briefly, cardiorespiratory fitness measured as VO_2_peak normalized with body mass (ml/kg/min) increased (P<0.001) in the E and SE groups: 20 (15) % and 16 (10) %, respectively. Furthermore, in MVC, significant (P<0.001) mean changes of 19 (17) % and 25 (21) % were observed in S and SE, respectively.

**Figure 2:**
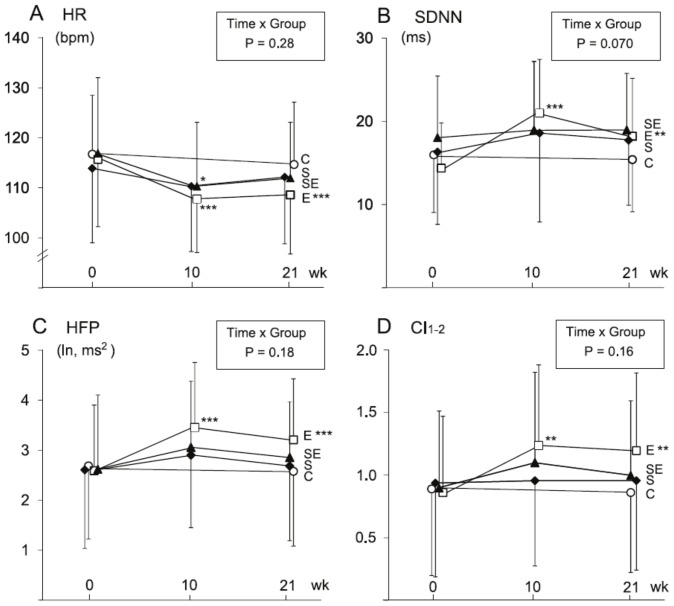
Heart rate and heart rate dynamics during steady state exercise. Heart rate (HR, A), standard deviation of NN intervals (SDNN, B), high frequency power (HFP, C) and HR complexity index for the scales 1 to 2 (CI_1–2_, D) measured at baseline (0) and after 10 and 21 weeks of endurance (E), strength (S) or combined strength and endurance training (SE) and in the control group (C). *significant change (P<0.05) compared to the baseline, **P<0.01, ***P<0.001.

**Figure 3:**
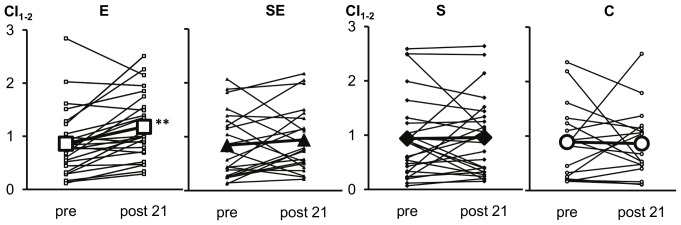
Individual and the mean changes in the HR complexity index. The index for the scales 1 to 2 (CI_1–2_) was measured during steady state exercise at baseline (pre) and after 21 weeks in the endurance training (E), combined strength and endurance training (SE), strength training (S) and the control (C) group. **significant change (P<0.01) compared to the baseline.

Significant time and group interactions were not observed among HR indices. Resting HR dynamics did not demonstrate training-induced changes in any of the groups (table 2). However, during steady state exercise the E group showed training-induced changes in HR dynamics (Fig. 2, table 3). Submaximal HR was significantly decreased, while SDNN, HFP and HR complexity (CI_1–2_) were significantly increased. In the SE group, mean HR was decreased after 10 weeks (Fig. 2A), but no other significant changes in HR dynamics were observed either after 10 or 21 weeks of combined training. Rather, the subjects in the SE group demonstrated an inconsistent change in HR dynamics compared to those in the E group (Fig. 3).

**Table 2:** Mean (95% confidence interval) for selected HRV indices at rest before (pre), and after 21 weeks of training (post) in the endurance, strength, combined strength and endurance training, and control group.

	Endurance	Strength	Combined	Control
HR, •min^−1^				
pre	63 (60 to 65)	62 (60 to 65)	62 (59 to 65)	65 (62 to 67)
post	60 (57 to 63)	61 (58 to 63)	62 (59 to 65)	62 (58 to 65)
SDNN, ms				
pre	50.2 (43.9 to 56.4)	53.4 (44.2 to 62.7)	51.3 (44.6 to 58.1)	45.1 (36.6 to 53.6)
post	49.3 (40.9 to 57.7)	57.0 (47.6 to 66.4)	48.0 (41.4 to 54.5)	47.0 (37.0 to 57.0)
HFP (ms^2^)				
pre	564 (351 to 778)	844 (316 to 1373)	574 (371 to 777)	431 (113 to 749)
post	616 (342 to 890)	1115 (446 to 1773)	494 (269 to 719)	563 (211 to 915)
LFP (ms^2^)				
pre	601 (436 to 767)	709 (437 to 982)	536 (422 to 650)	544 (272 to 817)
post	637 (393 to 881)	716 (424 to 1009)	494 (356 to 632)	654 (192 to 1116)
LFP/HFP				
pre	1.35 (0.99 to 1.71)	1.58 (0.56 to 2.60)	1.50 (0.96 to 2.04)	1.70 (1.29 to 2.11)
post	1.29 (0.86 to 1.72)	1.27 (0.77 to 1.77)	1.54 (0.97 to 2.10)	1.67 (0.78 to 2.55)
CI_1–5_				
pre	8.3 (7.7 to 9.0)	8.5 (7.6 to 9.5)	8.7 (8.0 to 9.4)	7.9 (6.9 to 8.9)
post	8.4 (7.2 to 9.6)	9.0 (7.7 to 10.3)	8.0 (7.2 to 8.9)	8.4 (7.1 to 9.7)

HR, heart rate; SDNN, standard deviation of NN intervals; HFP, high frequency power; LFP low frequency power; LFP/HFP, ratio between LFP and HFP; CI_1–5_, complexity index of multiscale entropy analysis over the scales of 1 to 5. Statistically significant changes were not observed.

**Table 3:** Mean change (including 95% confidence interval) for selected HRV indices measured during exercise.

	Endurance	Strength	Combined	Control
HR, •min^−1^				
Δ	−6.8 (−10.3 to −3.4)***	−1.7 (−5.9 to 2.5)	−4.8 (−10.3 to 0.7)	−2.1 (−7.8 to 3.6)
SDNN, ms				
Δ	4.6 (1.9 to 7.4)**	1.4 (−0.4 to 3.2)	0.7 (−2.9 to 4.3)	−0.6 (−4.6 to 3.4)
HFP (ln, ms^2^)				
Δ	0.65 (0.34 to 0.96)***	0.08 (−0.42 to 0.58)	0.23 (−0.38 to 0.84)	−0.11 (−0.84 to 0.63)
CI_1–2_				
Δ	0.32 (0.14 to 0.49)**	0.02 (−0.21 to 0.25)	0.10 (−0.14 to 0.35)	−0.03 (−0.40 to 0.34)

Comparison is done between the baseline and after 21 weeks of endurance training, strength training, combined strength and endurance training, and control period.

HR, heart rate; SDNN, standard deviation of NN intervals; HFP, high frequency power; CI_1–2_, complexity index of multiscale entropy analysis over the scales of 1 to 2.

** Statistically significant change at *P*<0.01,

*** *P*<0.001.

When examining the correlation coefficients between the changes in performance and the changes in HR indices, significant correlations were found in the resting condition between ΔMVC and ΔHR in S (r = −0.64, P<0.001) and between ΔVO_2_peak and ΔLFP/HFP in SE (r = 0.58, P = 0.006). In the exercise condition, significant correlations were found between ΔMVC and ΔHR in S (r = −0.40, P = 0.041) and between ΔVO_2_peak and ΔHR in SE (r = −0.47, P = 0.032). Significant correlations were not found in the E group. Training group specific correlations between the baseline and the change were found in most but not all HR indices (see tables S1 and S2 in the supporting information).

## Discussion

The main findings of the study were that the employed endurance training protocol led to 1) increases in both HR complexity and HRV in a group of healthy middle-aged women whereas strength training by itself or combined strength and endurance training did not produce significant changes in HR dynamics, and 2) endurance training-induced adaptations in HR dynamics were observed during steady state exercise but not during supine rest.

### Time Course of the Training Adaptations

The present endurance training program led to increases in HR complexity and HRV especially during the first 10 weeks of training. Furthermore, both endurance and combined strength and endurance training induced significant decreases in submaximal HR especially during the first ten weeks of training without further decreases during the latter half of the training period. The time course of the adaptations in submaximal HR was similar to what we have earlier reported in men (40 to 67 years) performing an identical training program [26].

It is possible that the present endurance training program was not sufficient to produce further improvements in HR dynamics during the latter half of training, even though both exercise intensity and training volume increased progressively throughout the training period. Training frequency, however, remained rather low and unaltered including only two weekly endurance training sessions. Based on earlier findings, even a prolonged training period may not necessarily lead to notable gains in HRV if the training dose is too low [29]–[31]. On the other hand, with three to five endurance training sessions per week at moderate or high intensity (70–90% of maximal HR), a considerably shorter training period (8–12 weeks) may produce significant changes in HRV both during exercise and at rest [32], [33].

### HR Dynamics after Combined Strength and Endurance Training

Strength training did not lead to significant changes in HR dynamics. Furthermore, endurance training combined with strength training did not increase HR complexity or HRV in this group of previously untrained middle-aged women. Although training-induced changes in the E group were not significantly different from those of the SE group, the inconsistent change observed in HR complexity raises the possibility that combined training for strength and endurance, with certain type of training programs and in certain individuals, may interfere with endurance training-induced adaptations in HR dynamics. Heterogeneity of responses may also increase when different training modes are combined compared to only performing endurance or strength training. We have shown previously in terms of VO_2_peak and maximal strength that the same subjects may not be systematically low or high responders to both endurance and strength training when combining the training modes [17]. The heritability of training responses [34] further suggests the possibility that genomic factors may partly determine whether the adaptations to endurance or strength training become more prominent.

Interestingly, the change in both resting and submaximal HR was associated with the change in MVC in the S group but not in the SE group. As the correlations were only modest and the mechanism linking the change in MVC and HR is unknown, caution should be exercised when interpreting these results. Speculatively, the mechanism responsible for the minor, non-significant decrease in HR in the S group may have been inhibited in the SE group. On the other hand, the mechanisms responsible for the significant changes in HR dynamics in the E group may also have been inhibited in the SE group. Thus, the SE group did not show a response that was a sum of the two training modes but rather an intermediate response.

We cannot conclude, however, whether strength training as such or the higher training volume in the SE group compared to the E group led to the inconsistent changes. Indeed, the individual changes in the CI_1–2_ values indicate that HR complexity was markedly increased in some subjects. Our previous findings in middle-aged men with a similar training program showed that fractal scaling properties of HR were improved after combined strength and endurance training at supine rest [26]. Although most studies have not shown significant changes in HRV after strength training, some studies have reported significant improvements in HR complexity [11] and fractal scaling properties of HR [12] with strength training in young men with three weekly training sessions, thus supporting the notion of the positive effects of strength training on cardiac autonomic function. In the present middle-aged women, however, strength training induced no significant changes in HR dynamics with two weekly strength training sessions. Further studies are needed to clarify whether the conflicting findings are due to the subject group characteristics, complex interplay of different training design variables or the use of different indices of HR dynamics.

### HR Dynamics during Exercise

The training-induced changes in HR dynamics with the present short-term recordings were observed during exercise and not at rest. Martinmäki et al. [29] also reported significant effects of endurance training on HRV when measured during exercise but not when measured at supine rest. These findings raise the possibility that the acute response to mild to moderate exercise may provide information not available in the basal, resting state. Some previous studies have reported poor reproducibility of short-term indices of HRV at rest during both paced and spontaneous breathing [35], [36], but improved reproducibility during physical exercise, possibly due to the reduced effect of unwanted confounding factors such as mental and nutritional state [37]. Furthermore, at rest the cardiovascular system functions at variable fractions of its capacity, while acute exercise may more consistently stress the regulatory mechanisms [37]. Therefore, the measurement of HR dynamics during low intensity exercise may be especially applicable for detecting subtle training-induced effects with short-term recordings of RR intervals.

The training-induced enhancement in HR dynamics measured during exercise may be at least partly due to the lower relative exercise intensity after training resulting from the improved cardiorespiratory fitness. In the present E group, the exercise intensity of 50 W represented an average relative exercise intensity of 34 and 29% of maximal aerobic cycling power during the cardiorespiratory fitness test before and after training, respectively.

### Effects of Endurance Training on Cardiac Autonomic Function

The endurance training-induced increase in HFP during exercise suggests that the decrease in submaximal HR was mediated, at least in part, by enhanced vagal modulation of HR. During low intensity exercise, such as the present cycling power of 50 W, the vagal modulation can still be observed, whereas at exercise intensities above 50–60% of maximal oxygen consumption HR is mainly regulated by sympathetic modulation [37]. Furthermore, changes in entropy based HR complexity positively correlate with the changes in cardiac vagal modulation [38]. However, the decrease in submaximal HR may have additionally resulted from decreased sympathetic activity. Sympathetic HR control has been previously reported to decrease as a result of endurance training and thus, partially account for the decrease in submaximal HR [39]. Without a reliable, direct measurement of the function of the sympathetic nervous system, which was not practicable in this study, the role of this component of the autonomic nervous system cannot be exactly determined.

### Study Limitations

The present study design precluded separating the effect of added strength training from the effect of the larger training volume in the combined strength and endurance training group compared to the endurance training only group. Thus, the cause of the disparate adaptations in the E and SE groups in terms of HR dynamics remains speculative. Furthermore, the training volume was rather low in the groups performing either endurance or strength training which may account for the non-significant changes in HR dynamics at rest. Four weekly training sessions in the combined training group were considered as a reasonable, practical upper limit of the training volume for untrained middle-aged subjects over the present fully supervised training period of 21 weeks. The prolonged duration of the supervised training intervention was, however, an important strength of the present study, and the midterm measurements allowed the evaluation of the time course of the adaptations.

As we did not find significant changes in HR dynamics at rest, it is possible that the repeatability of the HR measures may have not been high enough to detect subtle training induced changes. However, the present study was not designed to examine the reliability of HRV methods by repeating the baseline measurements. Our study does suggest the potential importance of obtaining measurements at low workload, as a complementary type of baseline.

### Conclusions

The present endurance training program with two weekly training sessions was a sufficient stimulus for positive changes in HR complexity and traditional HRV when evaluated during low intensity exercise, but not when recorded during supine rest. These adaptations mainly occurred during the first ten weeks of endurance training, which emphasizes the rapidity of health benefits after the initiation of even low volume regular exercise. Strength training twice a week or a combination of strength and endurance training with a total of four training sessions per week did not lead to significant changes in HR dynamics. The combined training group demonstrated an inconsistent change in HR complexity indicating that simultaneous training for strength and endurance may interfere with the endurance training-induced adaptations to HR dynamics. The heterogeneity observed in the training responses in the combined endurance and strength training group in terms of cardiac autonomic function adds up to the growing evidence supporting the individualization of training prescription based on individual health and fitness targets. Further studies are needed to test the present findings to explore possible gender and age related factors, as well as underlying mechanisms, that may mediate the effects of different training regimens on HR dynamics.

## Supporting Information

Table S1 **Correlations between the baseline and the change in HR indices at the resting condition.**
(DOCX)

Table S2 **Correlations between the baseline and the change in HR indices at the exercise condition.**
(DOCX)
