# Supplementary material for: Heart Rate Dynamics after Combined Strength and Endurance Training in Middle-Aged Women: Heterogeneity of Responses
Source: PLoS One. 2013 Aug 27;8(8):e72664. doi: 10.1371/journal.pone.0072664 (PMC3754931; doi:10.1371/journal.pone.0072664)
Supplement: Table S1 — Correlations between the baseline and the change in HR indices at the resting condition. (DOCX) [file pone.0072664.s001.docx]

Table S1. Correlations between the baseline and the change in HR indices at the resting condition.

| E (n=26) | HR | SDNN | HFP | LFP | LFP/HFP | CI_1-5_ |
| --- | --- | --- | --- | --- | --- | --- |
| ∆HR | -0.39 (0.052) | |  |  |  |  |
| ∆SDNN |  | -0.49 (0.010) | |  |  |  |
| ∆HFP |  |  | -0.084 (0.68) | |  |  |
| ∆LFP |  |  |  | -0.44 (0.026) | |  |
| ∆LFP/HFP |  |  |  |  | -0.43 (0.028) | |
| ∆ CI_1-5_ |  |  |  |  |  | -0.32 (0.12) |
| S (n=26) |  |  |  |  |  |  |
| ∆HR | -0.42 (0.034) | |  |  |  |  |
| ∆SDNN |  | -0.47 (0.015) | |  |  |  |
| ∆HFP |  |  | 0.029 (0.89) | |  |  |
| ∆LFP |  |  |  | -0.47 (0.017) | |  |
| ∆LFP/HFP |  |  |  |  | -0.87 (<0.001) | |
| ∆ CI_1-5_ |  |  |  |  |  | -0.082 (0.69) |
| SE (n=21) |  |  |  |  |  |  |
| ∆HR | -0.52 (0.017) | |  |  |  |  |
| ∆SDNN |  | -0.52 (0.015) | |  |  |  |
| ∆HFP |  |  | -0.33 (0.15) | |  |  |
| ∆LFP |  |  |  | -0.51 (0.019) | |  |
| ∆LFP/HFP |  |  |  |  | -0.28 (0.22) | |
| ∆ CI_1-5_ |  |  |  |  |  | -0.37 (0.10) |

Values are correlation coefficients (and P-values). HR, heart rate; SDNN, standard deviation of NN intervals; HFP, high frequency power; LFP low frequency power; LFP/HFP, ratio between LFP and HFP; CI_1-5_, complexity index of multiscale entropy analysis over the scales of 1 to 5.
