# Supplementary material for: Heart Rate Dynamics after Combined Strength and Endurance Training in Middle-Aged Women: Heterogeneity of Responses
Source: PLoS One. 2013 Aug 27;8(8):e72664. doi: 10.1371/journal.pone.0072664 (PMC3754931; doi:10.1371/journal.pone.0072664)
Supplement: Table S2 — Correlations between the baseline and the change in HR indices at the exercise condition. (DOCX) [file pone.0072664.s002.docx]

Table S2. Correlations between the baseline and the change in HR indices at the exercise condition.

| E (n=26) | HR | SDNN | HFP | CI_1-2_ |
| --- | --- | --- | --- | --- |
| ∆HR | -0.55 (0.004) |  |  |  |
| ∆SDNN |  | -0.072 (0.73) |  |  |
| ∆HFP |  |  | -0.55 (0.003) |  |
| ∆ CI_1-2_ |  |  |  | -0.37 (0.066) |
| S (n=26) |  |  |  |  |
| ∆HR | -0.49 (0.011) |  |  |  |
| ∆SDNN |  | -0.45 (0.022) |  |  |
| ∆HFP |  |  | -0.47 (0.016) |  |
| ∆ CI_1-2_ |  |  |  | -0.44 (0.025) |
| SE (n=21) |  |  |  |  |
| ∆HR | -0.67 (0.001) |  |  |  |
| ∆SDNN |  | -0.60 (0.004) |  |  |
| ∆HFP |  |  | -0.70 (<0.001) |  |
| ∆ CI_1-2_ |  |  |  | -0.48 (0.027) |

Values are correlation coefficients (and P-values). HR, heart rate; SDNN, standard deviation of NN intervals; HFP, high frequency power; CI_1-2_, complexity index of multiscale entropy analysis over the scales of 1 to 2.

­­
